# Evaluation of Commercially Available Serologic Diagnostic Tests for Chikungunya Virus

**DOI:** 10.3201/eid2012.141269

**Published:** 2014-12

**Authors:** Christine M. Prat, Olivier Flusin, Amanda Panella, Bernard Tenebray, Robert Lanciotti, Isabelle Leparc-Goffart

**Affiliations:** French Armed Forces Biomedical Research Institute (IRBA), Marseille, France (C.M. Prat, O. Flusin, B. Tenebray, I. Leparc-Goffart);; Centers for Disease Control and Prevention, Fort Collins, Colorado, USA (A. Panella, R. Lanciotti)

**Keywords:** arboviruses, Chikungunya virus, diagnostic tests, viruses

## Abstract

Chikungunya virus (CHIKV) is present or emerging in dengue virus–endemic areas. Infections caused by these viruses share some common signs/symptoms, but prognosis, patient care, and persistent symptoms differ. Thus, accurate diagnostic methods are essential for differentiating the infections. We evaluated 4 CHIKV serologic diagnostic tests, 2 of which showed poor sensitivity and specificity.

Disease caused by chikungunya virus (CHIKV), a mosquitoborne arbovirus (family *Togaviridae* family, genus *Alphavirus*), is clinically characterized by sudden-onset fever and severe arthralgia, which may persist for weeks, months, or years after the acute phase of the infection (*1*). Other symptoms of CHIKV infection (headache, fatigue, and rash) are common among many arboviral infections, including dengue.

CHIKV is endemic to some parts of Africa and causes recurrent epidemic waves in Asia and the Indian subcontinent. In 2005, CHIKV emerged in the Indian Ocean region (*2*), and at the end of 2013, the virus emerged in the Americas. The latter emergence occurred on St. Martin Island in the Caribbean, where autochthonous cases were confirmed in early December 2013; thereafter, the virus rapidly expanded to neighboring islands and territories (*3*). *Aedes* *aegypti* and *Ae. albopictus* mosquitoes, the vectors of CHIKV and dengue virus (DENV), are established in tropical and temperate regions of the world. The vulnerability of Europe to transmission of CHIKV and other arboviruses has been shown: autochthonous cases of CHIKV infection occurred in Italy in 2007 (*4*) and in France in 2010 (*5*), and cases of autochthonous dengue occurred in France in 2010 and 2013 (*6*,*7*).

The rate of CHIKV and DENV co-infections during the recent epidemic of CHIKV infections on St. Martin was 2.8% (*8*). It can be challenging to differentiate clinically between CHIKV and DENV infections, but it is crucial to do so because prognosis and patient care differ for these diseases.

The increasing threat of CHIKV emergence in temperate regions and the need to anticipate possible outbreaks of CHIKV infection are presenting a challenge to the current level of diagnostic preparedness. In France, a National Public Health plan for stopping the spread of CHIKV and DENV has been developed. The plan calls for detecting possible infections by obtaining clinical samples from patients with suspected cases and using vector control measures if needed. The diagnostic strategy (*9*) is twofold: for serum collected 1–7 days after the onset of symptoms, real-time PCR is used to detect viral genome; and for serum collected >5 days after onset of symptoms, serologic techniques are used to detect IgM and/or IgG responses to the virus. Real-time PCR testing can differentiate between DENV and CHIKV infections; however, a certain proportion of infected persons seek medical care at a time when real-time PCR is no longer effective for diagnosis. Thus, we evaluated commercially available serologic test kits that could be used widely.

One serologic testing method is the indirect fluorescent antibody (IFA) technique. Although IFA tests have good sensitivity and specificity (*10*) for CHIKV, this method requires specific material that may not be available in diagnostic laboratories worldwide. Furthermore, a previous study showed variability in IFA results between laboratories (*11*). Thus, we focused our analysis on 2 other serologic CHIKV detection methods: ELISA and immunochromatography test for rapid detection (RDT).

## The Study

We evaluated 4 commercially available serologic tests that are approved for CHIKV testing by the European Commission. Two of the tests were RDTs for CHIKV IgM: SD Bioline Chikungunya IgM (Standard Diagnostics Inc., Yongin-si, South Korea) and *OnSite* Chikungunya IgM Combo Rapid Test (CTK Biotech Inc., San Diego, CA, USA). The 2 other tests were ELISAs for the detection of CHIKV IgM and IgG: Chikungunya IgM μ-capture ELISA and Chikungunya IgG Capture ELISA (both from IBL International, Hamburg, Germany) and Anti-Chikungunya Virus ELISA IgM test and Anti-Chikungunya Virus ELISA IgG test (Euroimmun, Lübeck, Germany).

We obtained 2 sets of serum samples for testing: panel A (23 samples) and panel B (30 samples). The samples had been submitted to the French Armed Forces Biomedical Research Institute (IRBA; Marseille, France) for arbovirus testing during 2005–2014. We chose the serum samples on the basis of their reactivity against CHIKV and other genetically or clinically related arboviruses.

Panel A was characterized in the laboratories of 2 National Reference Centers for Arboviruses by using in-house ELISAs as previously described (*12*,*13*) and a neutralization test (*14*). One reference laboratory was at IRBA and the other was at the Centers for Disease Control and Prevention (Fort Collins, CO, USA). Both laboratories used an ELISA positivity threshold that was 3 times the reactivity of a negative control serum against viral antigens. Results from the 2 laboratories were 100% concordant (Tables 1, 2).

**Table 1:** Results of serologic diagnostic testing of 23 serum samples (panel A) in a study evaluating the accuracy of commercially available CHIKV test kits*

Virus tested, sample no.	In-house ELISA			In-house CHIKV neutralization, IRBA and CDC§	Commercially available RDT			Commercially available ELISA		
					SD Bioline Chikungunya IgM¶	OnSite Chikungunya IgM Combo Rapid Test#		Chikungunya IgM/IgG Capture**		Anti-Chikungunya Virus ELISA IgM/IgG††
	IRBA†		CDC‡							

IgM	IgG	IgM	IgG	IgM	IgG	IgM	IgG
CHIKV														
IgG														
1	−	+		−	+	5,120	+	−		−	+		−	−
2	−	+		−	+	1,280	−	−		−	−		−	+
3	−	+		−	+	>320	+	+		−	+		+	+
IgM + IgG														
4	+	+		+	+	320	−	−		+	+		+	+
5	+	+		+	+	40	−	−		+	+		+	−
6	+	+		+	+	2,560	+	−		+	+		+	+
7	+	+		+	+	1,280	−	+		+	+		NA	NA
8	+	+		+	+	640	+	+		+	+		+	+
9	+	+		+	+	320	+	−		+	−		+	+
10	+	+		+	+	80	−	−		+	−		+	+
IgM														
11	+	−		+	−	<10	−	−		+	−		+	−
12	+	−		+	−	<10	−	−		+	−		+	−
13	+	−		+	−	80	−	−		+	−		+	−
DENV														
IgM + IgG														
14	−	−		−	−	<10	−	−		−	−		−	−
15	−	−		−	−	<10	−	−		−	−		−	−
Negative samples‡‡														
16	−	−		−	−	<10	−	−		−	−		−	−
17	−	−		−	−	<10	+	−		−	−		−	−
18	−	−		−	−	<10	−	−		-	−		−	−
19	−	−		−	−	<10	+	−		+	−		−	−
20	−	−		−	−	<10	−	−		+	−		+	NA
21	−	−		−	−	<10	−	−		−	−		−	−
22	−	−		−	−	<10	−	−		−	−		−	−
23	−	−		−	−	<10	−	−		−	−		−	−

*The serum samples were obtained from IRBA (Marseille, France). CDC, Centers for Disease Control and Prevention; CHIKV, chikungunya virus; DENV, dengue virus; IRBA, French Armed Forces Biomedical Research Institute; NA, not applicable; RDT, immunochromatography test for rapid detection. †National Reference Center for Arboviruses at IRBA. ‡National Reference Center for Arboviruses at CDC (Fort Collins, CO, USA). §Data are CHIKV neutralization titers of serum. ¶From Standard Diagnostics Inc., Yongin-si, South Korea. #From CTK Biotech Inc., San Diego, CA, USA. **From IBL International, Hamburg, Germany. ††From Euroimmun, Lübeck, Germany. ‡‡Depending on the patient’s recent travel history, these samples were tested for various other viruses. Test results were negative for following arboviruses: DENV, CHIKV, West Nile virus, Toscana virus, Japanese encephalitis virus, Rift Valley fever virus, St. Louis encephalitis virus, Mayaro virus.

**Table 2:** Results of serologic diagnostic testing of 23 serum samples (panel B) in a study evaluating the accuracy of commercially available CHIKV test kits*

Virus tested, sample no.	In-house ELISA			In-house CHIKV neutralization, IRBA and CDC§	Commercially available RDT			Commercially available ELISA		
								Chikungunya IgM/IgG Capture**		Anti-Chikungunya Virus ELISA IgM/IgG††
					SD Bioline Chikungunya IgM	OnSite Chikungunya IgM Combo Rapid Test#				
	IRBA†		CDC‡							

IgM	IgG		IgM	IgG	IgM	IgG	IgM		IgG
CHIKV															
IgG															
24	−	+		NA	NA	NA	NA	NA		−	−		+		+
25	−	+		NA	NA	NA	NA	NA		−	−		+		+
26	−	+		NA	NA	NA	NA	NA		−	−		−		+
27	−	+		NA	NA	NA	NA	NA		−	−		−		+
28	−	+		NA	NA	NA	NA	NA		−	−		−		+
29	−	+		NA	NA	NA	NA	NA		−	+		−		+
IgM + IgG															
30	+	+		NA	NA	NA	NA	NA		+	+		NA		NA
31	+	+		NA	NA	NA	NA	NA		+	+		+		+
32	+	+		NA	NA	NA	NA	NA		+	+		+		+
33	+	+		NA	NA	NA	NA	NA		−	−		+		+
34	+	+		NA	NA	NA	NA	NA		+	−		+		+
35	+	+		NA	NA	NA	NA	NA		+	−		+		+
36	+	+		NA	NA	NA	NA	NA		−	−		−		+
37	+	+		NA	NA	NA	NA	NA		+	+		+		−
38	+	+		NA	NA	NA	NA	NA		+	−		+		+
39	+	+		NA	NA	NA	NA	NA		+	+		+		+
40	+	+		NA	NA	NA	NA	NA		+	+		+		NA
41	+	+		NA	NA	NA	NA	NA		+	+		+		+
IgM															
42	+	−		NA	NA	NA	NA	NA		+	−		+		NA
43	+	−		NA	NA	NA	NA	NA		−	−		−		−
44	+	−		NA	NA	NA	NA	NA		−	−		+		−
45	+	−		NA	NA	NA	NA	NA		+	−		+		−
46	+	−		NA	NA	NA	NA	NA		−	−		−		−
47	+	−		NA	NA	NA	NA	NA		−	−		−		−
DENV															
IgM + IgG															
48	−	−		NA	NA	NA	NA	NA		−	−		−		−
49	−	−		NA	NA	NA	NA	NA		−	−		−		−
50	−	−		NA	NA	NA	NA	NA		−	−		−		+
RVRV‡‡															
IgG, 51	−	+		NA	NA	<10	NA	NA		−	−		NA		NA
MAYV‡‡															
IgM + IgG, 52	−	+		NA	NA	<10	−	−		−	−		−		+
ONNV‡‡															
IgM + IgG, 53	+	+		NA	NA	<10	−	−		+	+		+	+	

*The serum samples were obtained from IRBA (Marseille, France). CDC, Centers for Disease Control and Prevention; CHIKV, chikungunya virus; DENV, dengue virus; IRBA, French Armed Forces Biomedical Research Institute; MAYV, Mayaro virus; NA, not applicable; ONNV o’nyong-nyong virus; RDT, immunochromatography test for rapid detection; RVRV, Rift Valley fever virus. †National Reference Center for Arboviruses at IRBA. ‡National Reference Center for Arboviruses at CDC (Fort Collins, CO, USA). §Data are CHIKV neutralization titers of serum. ¶From Standard Diagnostics Inc., Yongin-si, South Korea. #From CTK Biotech Inc., San Diego, CA, USA. **From IBL International,Hamburg, Germany. ††From Euroimmun, Lübeck, Germany. ‡‡These samples were characterized by neutralization techniques against 4 viruses in parallel: RRV, MAYV, ONNV, and CHIKV.

Panel B was tested by using in-house techniques at IRBA. Because sample volumes were limited, we used panel A to test the commercial kits and used panel B only if the specificity and sensitivity of tests on panel A were >70%. Commercial tests were performed according to manufacturers’ protocols.

We used SD Bioline and CTK Biotech RDTs to process panel A samples plus serum samples (1 each) infected with Mayaro virus and o’nyong-nyong virus (Tables 1, 2). Neither Mayaro virus nor o’nyong-nyong virus was detected by the RDTs. The SD Bioline RDT showed poor sensitivity (30%) and specificity (73%) for CHIKV in panel A samples, and 39% and 57% of the results were false negative and false positive, respectively. The CTK kit showed 93% specificity and 20% sensitivity for CHIKV in panel A samples, and 36% and 33% of the results were false negative and false positive, respectively. The ineffectiveness of the RDT kits was demonstrated by panel A test results, so panel B was not tested.

### Commercially Available IgM and IgG ELISAs

We used chikungunya IgM/IgG ELISAs from Euroimmun and IBL International (Tables 1, 2) to process panel A samples plus serum samples (1 each) infected with Mayaro virus and o’nyong-nyong virus. The specificity and sensitivity of the ELISAs for this set of samples were >70%, so we also tested panel B.

ELISAs from both companies detected o’nyong-nyong virus IgM and IgG. The Euroimmun ELISA detected Mayaro virus IgG but not IgM; the IBL International ELISA did not detect Mayaro virus IgG or IgM. This cross-reactivity highlights the fact that seroneutralization is necessary to differentiate between viruses in the same serogroup. The IBL ELISA had a specificity of 88% (IgM) and 96% (IgG) and a sensitivity of 79% (IgM) and 52% (IgG). For IgM detection, 12% of the IBL ELISA results were false positive and 21% were false negative. The Euroimmun ELISA had a specificity of 82% (IgM) and 95% (IgG) and a sensitivity of 85% (IgM) and 88% (IgG). For IgM detection, 18% of the Euroimmun ELISA results were false positive and 15% were false negative.

## Conclusions

In our evaluation, the commercial RDTs that we compared with in-house ELISAs from 2 National Reference Centers for Arboviruses performed poorly. A previous evaluation study that used the same RDTs to process serum samples from residents of Indonesia had results in the same range as our results (*15*); together, these findings show that the kits should not be used in clinical settings, regardless of the geographic origin of the infection. The 2 ELISAs that we tested had better sensitivity and specificity than the RDTs; however, they had a non-negligible number of false-negative and false-positive results.

If the current outbreak of CHIKV infection in the Americas follows the same trend as that seen in the 2005 Réunion Island outbreak, increased circulation of the virus can be expected, and diagnostic laboratories must be prepared. A 2009 international evaluation of the diagnostic quality of 30 expert laboratories showed that most of the laboratories needed more sensitive CHIKV IgM detection assays; results for IgM were correct in only 50.7% of cases (*11*). Our evaluation was a pilot study using a small number of samples, but the findings show the importance of evaluating commercial diagnostic kits and published protocols before using such tools in clinical settings.
